# Enantioselective carbenoid insertion into C(sp^3^)–H bonds

**DOI:** 10.3762/bjoc.12.87

**Published:** 2016-05-04

**Authors:** J V Santiago, A H L Machado

**Affiliations:** 1 Grupo de Tecnologia em Síntese Orgânica, Instituto de Química, Universidade de Brasília, Campus Universitário Darcy Ribeiro, 4478, CEP 70904-970, Asa Norte, Brasília-DF, Brasil

**Keywords:** C–H activation, chiral catalysis, diazo compounds, total synthesis

## Abstract

The enantioselective carbenoid insertion into C(sp^3^)–H bonds is an important tool for the synthesis of complex molecules due to the high control of enantioselectivity in the formation of stereogenic centers. This paper presents a brief review of the early issues, related mechanistic studies and recent applications on this chemistry area.

## Introduction

One of the major challenges met in organic synthesis is the formation of carbon–carbon bonds, in particular in a stereoselective way. Nucleophilic substitution reactions, radical reactions, cross-coupling reactions and the Heck reaction are well-known approaches available to this goal. These reactions are based on the polar characteristic of the carbon–halogen or carbon–pseudohalogen bonds, as a result of the electronegativity difference between these atoms. Despite the proven success of these transformations, they are limited to pre-functionalization of the chemical structure of interest with halogen atoms or pseudohalogen functional groups.

One approach that has been gaining increasing attention, by not requiring the presence of a strongly polarized chemical bond, is the C(sp^3^)–H bonds activation by carbenoids [1]. The enantioselective insertion of these organometallic species into these non-polarized bonds is a recent topic in the chemical literature, when compared to the first reports of carbenoid chemistry around the 1950s.

**Figure 1:**
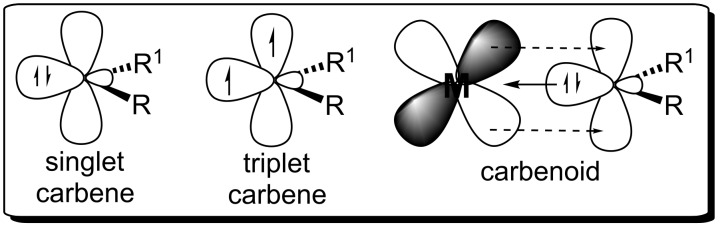
Singlet carbene, triplet carbene and carbenoids.

Carbene is a molecule bearing a functional group with a divalent neutral carbon. This structural framework results in the presence of a nonbonding electron pair that may adopt two electronic configurations: singlet and triplet (Figure 1). A carbenoid is an organometallic complex where the carbene acts as a neutral ligand to a metal center. This ensures a greater stability of the carbene, allows the modulation of its reactivity, and controls the chemo-, regio- and stereoselectivity in reactions.

The activation of the C(sp^3^)–H bond needs an appropriate interaction between the carbenoid intermediate and the carbon atom of the C(sp^3^)–H. Depending on the electronic demand of the substituent attached to the carbene carbon atom, the insertion reaction can be more or less selective. Very electrophilic carbenoid intermediates, for example, display little regio- and stereoselectivity, favoring the occurrence of side reactions. A less electrophilic carbenoid intermediate, on the other hand, has a lower reactivity, but its regio- and stereoselectivity are better [2].

**Figure 2:**
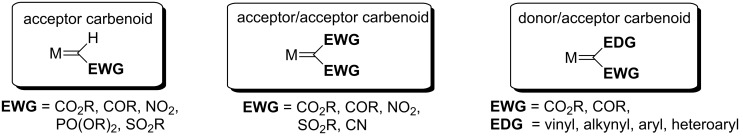
Classification of the carbenoid intermediates by the electronic nature of the groups attached to the divalent carbene carbon.

The electrophilicity of the carbenoid intermediate is related to the substituents present in its structure. Electron-withdrawing groups (EWGs) increase the electrophilicity of the carbon atom in the carbenoid and electron-donating groups (EDGs) act with the opposite effect. Due to these observations, a carbenoid intermediate can be divided in three different classes based on the electronic nature of the chemical groups attached to its structure: acceptor, donor/acceptor and acceptor/acceptor (Figure 2) [2]. The modification of the substituents on the carbenoid intermediate can change its reactivity and hence the selectivity of the carbenoid reaction.

The most commonly used diazo compounds rely on the formation of a donor/acceptor carbenoid intermediate type. The EWG increases the electrophilicity and reactivity of the donor/acceptor carbenoid, while an EDG increases its stability and selectivity [2].

Despite the importance of the electronic factors to the reactivity and selectivity of carbenoid intermediates, steric and conformational effects are also determining factors for carbenoid chemistry. Steric as well as electronic factors and the chemical properties of the ligands around the metal center also determine significantly the type of insertion performed by the carbenoid intermediate. The complexes used for the formation of carbenoids in enantioselective insertion reactions must present a balance between steric and electronic factors, to promote the formation of a specific enantiomer.

The search for the best balance of these properties of the carbenoid intermediates was also sought through the use of different metals such as copper [3], rhodium [4], iron [5], ruthenium [6], iridium [7], osmium [8], and others. From these, copper and rhodium have been the most frequently used ones in carbenoid insertion reactions.

**Figure 3:**
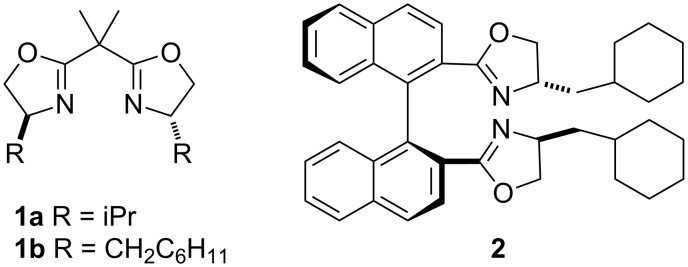
Chiral bis(oxazoline) ligands used in enantioselective copper carbenoid insertion.

Copper carbenoids having a higher electrophilic character display a great reactivity, but little selectivity in insertion reactions. Despite these features, only recently the insertion of chiral copper carbenoids into a C(sp^3^)–H bond has gained special attention, as in the works of Muler and Boléa [9], Flynn [10], Stattery [11] and their respective co-workers. The most selective copper carbenoids are those generated from chiral bis(oxazoline) ligands in the presence of copper(I) triflate (CuOTf) (Figure 3).

The rhodium carbenoid intermediates are prferably used in enantioselective insertion reactions. They are more often found as dirhodium(II) complexes. Only one of the two metal atoms present in the chemical structure effectively participates in the insertion reaction. The other rhodium atom withdraws electron density from the rhodium atom involved in the insertion mechanism increasing therefore its electrophilic character [12]. Four specific types of chiral rhodium(II) complexes can be found as catalyst in enantioselective insertion reactions of carbenoids in C(sp^3^)–H bonds: carboxylates [13–19], carboxamides [20–23], phosphates [24–25], and ortho-arylphosphines [26–30].

This work aims to review the chemical literature, since 2009 [31–32] until the end of 2015, concerning the development of catalytic systems able to promote enantioselective carbenoid insertion into C(sp^3^)–H bonds, the mechanistic aspects recently discovered to the known catalytic systems and the application of these synthetic tools to the organic synthesis of natural products.

## Review

### Historic perspective on the carbenoid reaction insertion into X–H bonds

**Scheme 1:**
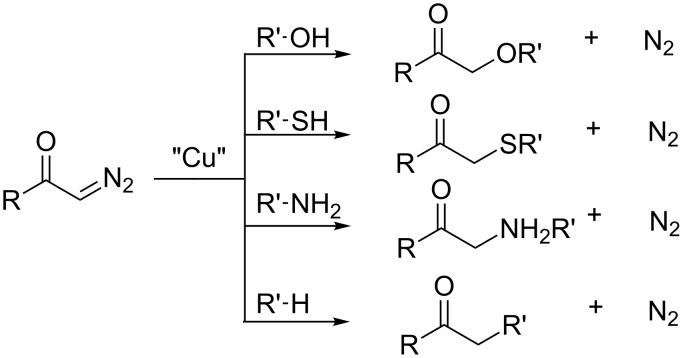
Pioneering work of Peter Yates on the carbenoid insertion reaction into X–H bonds (where X = O, S, N, or C).

One of the former works to address the chemistry of carbenoid was reported in 1952 by Peter Yates, although the author does not specifically use the term carbenoid, but – carbene–copper complex (Scheme 1) [3]. In his opinion, the copper catalyst promotes the decomposition of diazoketones to afford "free carbenes", the chemical intermediates responsible for the insertion reaction in X–H bonds (X = O, S, N, or C).

**Scheme 2:**
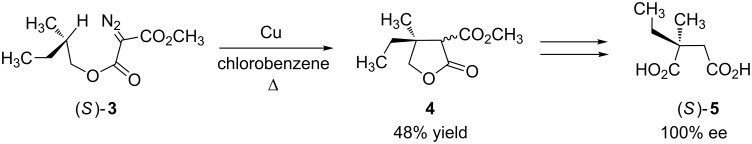
Copper carbenoid insertion into C(sp^3^)–H bond of a stereogenic center with full retention of the asymmetric carbon configuration.

Ledon et al, in 1973, showed a very important feature of the carbenoid insertions into C(sp^3^)–H bonds. The intramolecular reaction of the chiral diazomalonate (*S*)-**3** led to the insertion of the carbenoid intermediate into the C(sp^3^)–H of the stereogenic center with full retention of the asymmetric carbon configuration (Scheme 2) [33].

The authors demonstrated that an insertion reaction in C(sp^3^)–H bonds only occurs with considerable yield when small amounts of copper powder or copper salts, such as CuSO_4_ and CuCN, were employed. Even using the term "carbenoid", the work does not present the formation of a copper carbenoid intermediate. It only suggests an insertion reaction occurring through free carbenes with copper working only as a catalyst to promote the carbene formation.

**Scheme 3:**
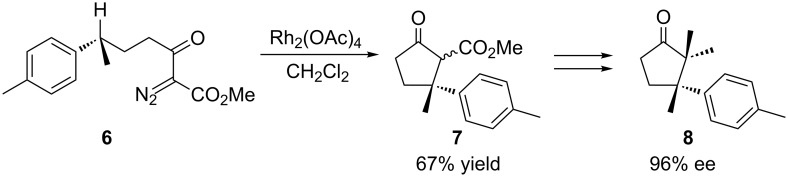
Carbenoid insertion into a C(sp^3^)–H bond as the key step of the Taber’s (+)-α-cuparenone (**8**) synthesis.

In 1985, Taber and coworkers reported the synthesis of (+)-α-cuparenone (**8**) through the construction of a five-membered ring prepared by an enantioselective carbenoid insertion into a C(sp^3^)–H bond (Scheme 3) [34]. To carry out the cyclization, the carbenoid was formed by the action of Rh_2_(OAc)_4_ on the diazo compound **6**. That intermediate intramolecularly inserted into the C(sp^3^)–H bond of the asymmetric carbon to yield ketoester **7** in 67% yield. This latter compound was converted to (+)-α-cuparenone (**8**) in 26% yield and 96% enantiomeric excess.

In the late 1980s, many studies have been published by Taber [35], Sonawane [36], Doyle [37] and their respective coworkers regarding the regiochemistry of carbenoid insertion into C(sp^3^)–H bonds, and also the steric and electronic factors related to this insertion.

**Scheme 4:**
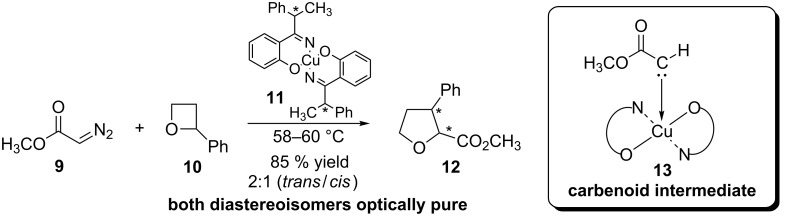
First enantioselective carbenoid insertion into C–O bonds catalyzed by chiral metallic complexes.

The first example of an enantioselective carbenoid insertion reaction in chemical bonds catalyzed by chiral metal complexes was introduced in 1966 by Noyori and coworkers (Scheme 4) [38]. In addition to the novelty of the use of the chiral copper complex **11** for controlling the enantioselectivity, the authors proposed the participation of the copper carbenoid **13**, formed from the reaction between the copper complex **11** and methyl diazoacetate **9** as active intermediate in the catalytic cycle of this transformation.

From the 1990s, the enantioselective carbenoid insertion into C(sp^3^)–H bonds starts to be better discussed in the literature. Ikegami and coworkers reported the enantioselective insertion of α-diazo-β-ketoesters into C(sp^3^)–H bonds catalyzed by rhodium carboxylate complexes in their homochiral form (Table 1) [39]. Modest enantiomeric excesses were provided by the three tested catalysts. The reactions carried out with complex **17a** and **17b** show very similar stereoselectivity, forming the *R*-enantiomer of compound **16** as the main product after decarboxylation reaction. The catalyst **17c** showed opposite enantioselectivity when compared to the catalysts **17a** and **17b**, with the *S*-enantiomer formed as the major product.

**Table 1:** Enantioselective insertion of α-diazo-β-ketoesters into C(sp^3^)–H bonds catalyzed by chiral rhodium(II) complexes **17a**, **17b** and **17c**.

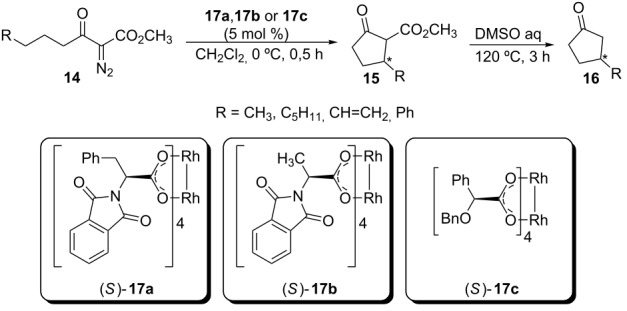				

R	catalyst	yield (%)	ee (%)	configuration of **16**

CH_3_	**17a**	76	24	*R*
CH_3_	**17b**	73	24	*R*
CH_3_	**17c**	75	10	*S*
C_5_H_11_	**17a**	43	29	*R*
CH=CH_2_	**17a**	44	38	*R*
CH=CH_2_	**17b**	39	35	*R*
CH=CH_2_	**17c**	44	30	*S*
Ph	**17a**	96	46	*R*
Ph	**17b**	87	43	*R*
Ph	**17c**	73	13	*S*

**Figure 4:**
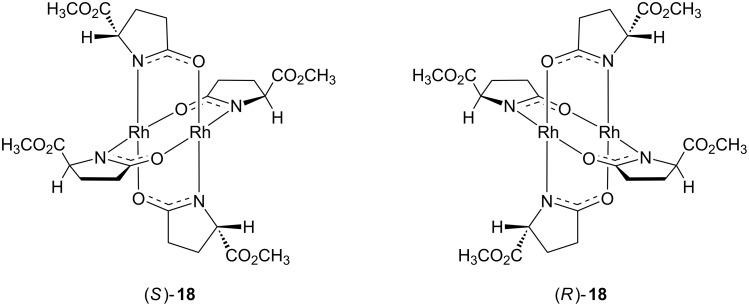
Chemical structures of complexes (*R*)-**18** and (*S*)-**18**.

In 1991, Doyle and coworkers published asymmetric synthesis of lactones from alkyl diazoacetates in high enantioselectivity by intramolecular rhodium carbenoid insertion into C(sp^3^)–H [40]. In this work, the authors introduced the enantiomeric rhodium(II) carboxamides complexes (*R*)-**18** and (*S*)-**18** (Figure 4).

The authors could observe the enantioselective formation of the lactones **20** with high enantiomeric excess (Table 2). The carbenoid formed by (*S*)-**18** favored the *S* configuration at the generated stereogenic center for most of the prepared lactones. The opposite preference, *R* configuration at the new stereogenic center of **20**, was reported to the use of the enantiomeric rhodium complex (*R*)-**18**. When substrate **19f** reacts under catalysis of rhodium(II) carboxamide complexes (*R*)-**18** and (*S*)-**18**, the configuration of the new stereogenic center of **20f** was reversed, probably due to the lack of the oxygen atom in the substituent R, as suggested by the authors.

**Table 2:** Enantioselective intramolecular insertion of carbenoids into C(sp^3^)–H bonds catalyzed by rhodium (II) carboxamides complexes (*R*)-**18** and (*S*)-**18**_._

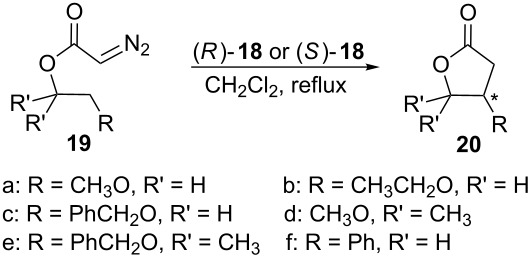				

Reagent	catalyst	yield (%)	ee (%)	configuration of **20**

**19a**	(*S*)-**18**	62	91	*S*
**19a**	(*R*)-**18**	73	91	*R*
**19b**	(*S*)-**18**	64	89	*S*
**19b**	(*R*)-**18**	63	89	*R*
**19c**	(*S*)-**18**	64	87	*S*
**19c**	(*R*)-**18**	69	87	*R*
**19d**	(*S*)-**18**	68	56	*S*
**19d**	(*R*)-**18**	70	57	*R*
**19e**	(*S*)-**18**	85	51	*S*
**19f**	(*S*)-**18**	42	46	*R*
**19f**	(*R*)-**18**	34	45	*S*

**Scheme 5:**
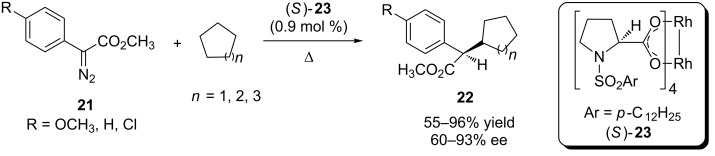
Asymmetric carbenoid insertions into C(sp^3^)–H bonds of cycloalkanes catalyzed by chiral rhodium carboxylate complexes.

In 1997, Davies and Hansen reported the intermolecular carbenoid insertion into C(sp^3^)–H catalyzed by rhodium complex (*S*)-**23** with good to excellent enantioselective control (Scheme 5) [41]. The best results were observed when the reaction was carried out at room temperature. A wide range of substituent groups were evaluated at *para* position of the aryldiazoacetate aromatic ring. The cyclic hydrocarbon reagents, also used as solvent, were cyclopentane, cyclohexane and cycloheptane.

Two factors are noteworthy in this work. Unlike the carboxamide complexes (*R*)-**18** and (*S*)-**18** previously reported by Doyle and coworkers (Table 2), where the complexation of the chiral ligand to rhodium atoms occurs through the carboxamide group, in the new chiral catalyst (*S*)-**23** the rhodium atoms are complexed to the chiral ligands by the carboxylate group, similar to those chiral complexes presented by Ikegami and coworkers (Table 1). Another important feature of this work is, unlike to the work that preceded it, that the new stereogenic center is formed on the carbenoid carbon coordinated to the metal rhodium center and not on carbon-containing the C(sp^3^)–H bond activated by the carbenoid moiety.

**Scheme 6:**
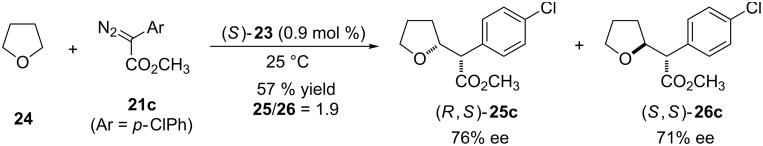
First diastereo and enantioselective intermolecular carbenoid insertion into tetrahydrofuran C(sp^3^)–H bond.

The authors also reported in this work the insertion into the C(sp^3^)–H bond of tetrahydrofuran. This reaction showed good yield, regio-, diastereo- and enantioselectivity and represents the first example of the formation of a new stereogenic center out of the diazoacetate scaffold by an intermolecular carbenoid insertion into C(sp^3^)–H bond (Scheme 6).

### Mechanism of the carbenoid insertion into the C(sp^3^)–H bond

**Scheme 7:**
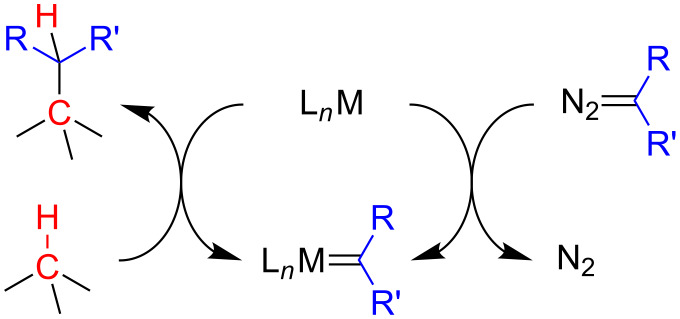
Simplified mechanism of the carbenoid insertion into a C(sp^3^)–H bond.

Nakamura [12] and Doyle [37] were the first to do important contributions to the comprehension of the mechanism of this catalytic cycle. In a simplified form, the mechanism of the carbenoid insertion into a C(sp^3^)–H bond can be represented as outlined in Scheme 7.

**Scheme 8:**
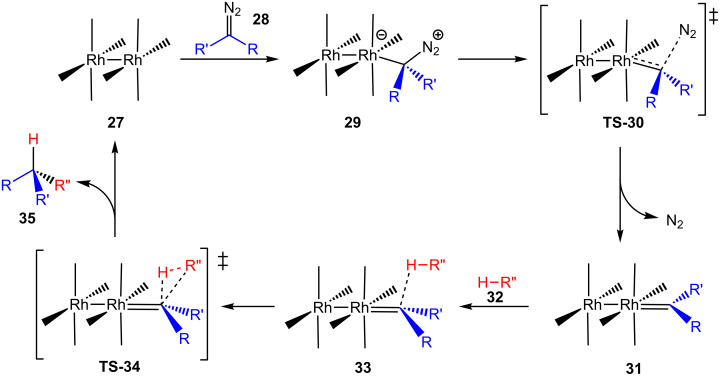
Nakamura’s carbenoid insertion into a C(sp^3^)–H bond catalytic cycle.

Nakamura et al. investigated the dirhodium tetracarboxylate-mediated carbenoid insertion reaction into C(sp^3^)–H bonds in more detail using the relationship between the transition-state structures and their corresponding free energies obtained by DFT investigation (Scheme 8) [12]. The insertion step primarily consists in the formation of the metal carbenoid **29** by the interaction of the diazo compound **28** and the dirhodium complex **27**. In sequence, the reaction proceeds through the transition state **TS-30** to release N_2_, and yields the carbenoid **31**. The divalent carbon attached to the rhodium atom starts to interact with the hydrogen of the C(sp^3^)–H bond of the compound **32** to form the van der Waals complex **33** which undergoes through the transition state **TS**-**34** to the product of the carbenoid insertion reaction **35**, regenerating the dirhodium complex **27**.

**Scheme 9:**
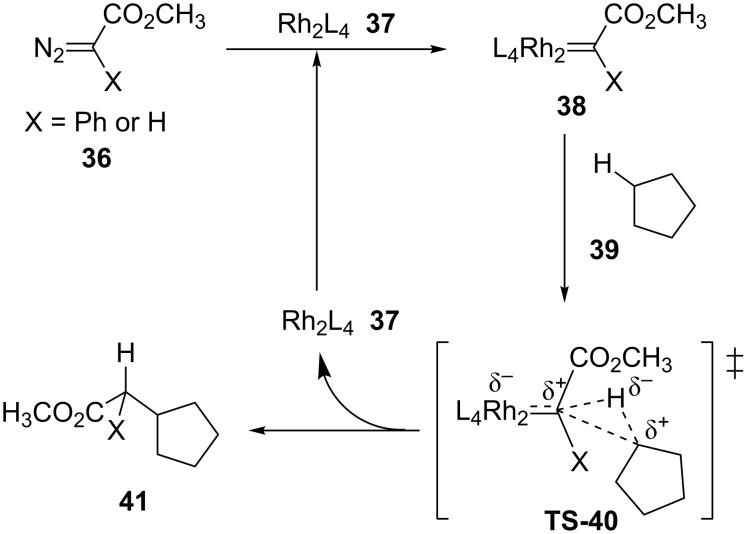
Investigation of the relationship between the electronic characteristics of the substituent X attached to the carbenoid divalent carbon and the selectivity of the carbenoid insertion into σ C(sp^3^)–H bonds.

In 2009, Davies and coworkers reported a DFT investigation of the relationship between the electronic characteristics of the substituent X attached to the carbenoid divalent carbon and the selectivity toward carbenoid insertion into σ C(sp^3^)–H bonds (Scheme 9) [42].

**Scheme 10:**
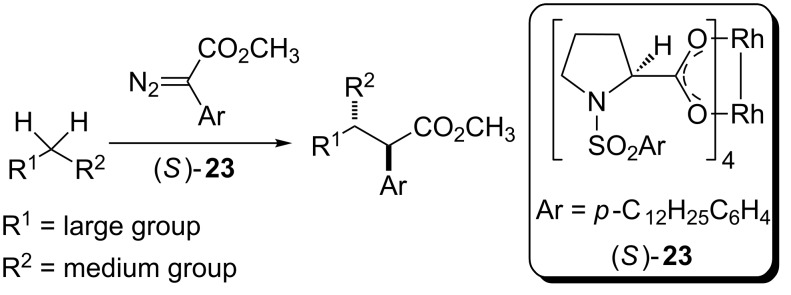
Empirical model to predict the stereoselectivity of the donor/acceptor dirhodium carbenoid insertion into C(sp^3^)–H catalyzed by (*S*)-**23**.

The authors found an exergonic carbenoid formation step and proposed two reasons for the selectivity toward insertion of this carbenoid into σ C(sp^3^)–H when X = Ph or H. The first reason concerns the relative stability of the carbenoids **38-Ph** and **38-H**. The first one, prepared from the donor/acceptor diazo compound **36-Ph**, is 10.9 kcal more stable than the carbenoid **38-H** obtained from the acceptor diazo compound **36-H**. This observation was attributed to the stabilization of the partial positive charge on the divalent carbon of the transition state **TS-40** provided by the phenyl donor group. The second reason concerns the large difference between the activation energy of these reactions which relies on the development of steric strain through the transitions state **TS-40**, less important when X = H. This study provided a simple empirical model able to predict the stereoselectivity of the intermolecular insertion of donor/acceptor dirhodium carbenoids into C(sp^3^)–H bonds properly validated by the results obtained by this research group during the development of the chiral catalyst (*S*)-**23** (Scheme 10).

### Recent studies concerning the enantioselective carbenoid insertion into C(sp^3^)–H bonds

From 2000, the study of carbenoid chemistry has become more comprehensive. The focus of most recently published works is the development of new catalysts for carbenoid insertion reactions into C(sp^3^)–H bonds and also the insertion into X–H bonds, where X = N, O, S, Si and others.

#### Copper-based chiral catalysts

In 2002, Müller and Boléa published a study evaluating the enantioselective insertion of copper carbenoids formed from phenyliodonium ylides and diazo compounds (Table 3) [9]. This work is particularly important because, at that time, the carbenoids derived from rhodium complexes were the most used for insertion reactions in C(sp^3^)–H bonds.

**Table 3:** Enantioselective intramolecular insertion of copper carbenoids derived from phenyliodonium ylides and diazo compounds.

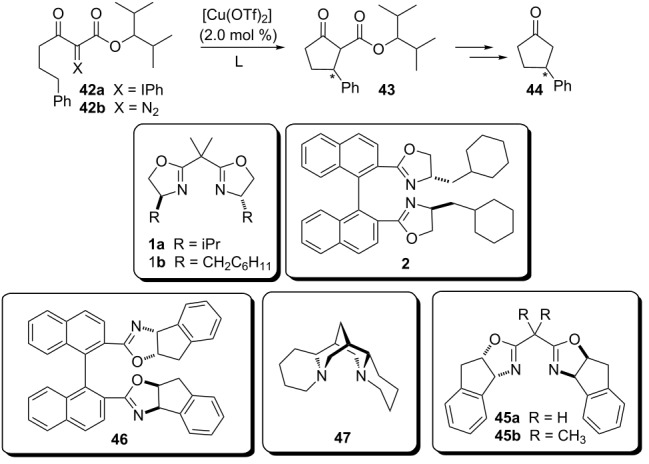				

L^a^	yield (%) from 42a^a^	ee (%)	yield (%) from **42b**^b^	ee (%)

**1a**	51	(*R*)-**42**	38	(*R*)-**15**
**1b**	49	(*R*)-**38**	32	(*R*)-**18**
**45a**	52	(*S*)-**72**	14	(*S*)-**31**
**45b**	46	(*S*)-**17**	55	(*S*)-**22**
**2**	46	(*R*)-**59**	35	(*R*)-**60**
**46**^c^	47	(*S*)-**67**	17	(*S*)-**51**
**47**	11	(*S*)-**57**	32	(*S*)-**18**

^a^CH_2_Cl_2_ at 0 °C; ^b^ClCH_2_CH_2_Cl at 65 °C; ^c^Ligand **46** was used in 70% de. When **42a** was cyclized by **46** with de > 98%, the ee of the product **43** increased to 70% ee favoring the same stereoisomer (*S*).

Comparing the results of Table 3, the same enantiomer was obtained mainly for both carbenoid precursors, ylide **42a** and the diazo compound **42b.** The authors suggested the formation of the same chiral copper carbenoid intermediate by the reaction of the in situ prepared chiral copper complexes with both **42a** and **42b** to provide the observed insertion products.

The reactions with phenyliodonium ylides **42a** showed better ee when compared to that done with diazo compounds **42b**. The authors attributed this observation to the large difference between the reaction temperatures, 0 °C to ylides versus 65 °C to diazo compounds. Higher temperatures increase the carbenoid formation rate by the chiral copper complexes as well as the carbene formation rate by direct decomposition of the precursors **42a** and **42b**. The competition between carbene insertion and chiral carbenoid insertion into C(sp^3^)–H bonds decreases the enantioselectivity of this transformation.

To confirm the copper carbenoid formation and its participation on the insertion reactions, the authors prepared the carbenoid precursors (*R*)-**48a** and (*R*)-**48b** and submit them to Rh_2_(OAc)_4_ or Cu(hfa)_2_ catalysis (Table 4). All reactions maintained the configuration of the asymmetric carbon where de insertion happened, independent to the carbenoid precursor and the catalyst, a strong evidence of the carbenoid intermediates formation.

**Table 4:** Experimental evidences of the carbenoid formation from (*R*)-**48a** and (*R*)-**48b** and its intramolecular insertion into C(sp^3^)–H bonds.

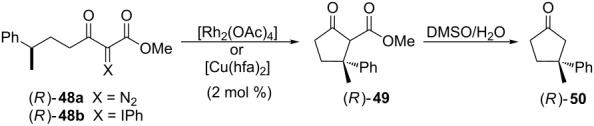				

precursor	catalyst	time	(*R*)-**49** yield (%)	ee (%)

(*R*)-**48a**	[Rh_2_(OAc)_4_]	30 min	59	>98
(*R*)-**48a**	[Cu(hfa)_2_]	3h	54	>98
(*R*)-**48b**	[Rh_2_(OAc)_4_]	3h	57	>98
*(R*)-**48b**	[Cu(hfa)_2_]	3h	36	>98

In 2010, Maguire et al. studied the enantioselective insertion of copper carbenoid derived from α-diazosulfones into C(sp^3^)–H bonds [10]. In this work, the authors produced cyclic sulfones (thiopyrans) **52** with high enantioselectivity by using a combination of 5 mol % of copper chloride salt, 6 mol % of ligand **1c** and 6 mol % of sodium tetrakis[(3,5-tri-fluoromethyl)phenyl]borate (NaBARF). The cyclic sulfones **52** were obtained in good yields and excellent enantiomeric excesses (85–98%) favoring the *cis*-1,2-di-substituted stereoisomer (Table 5).

**Table 5:** Insertion of asymmetric copper carbenoid C(sp^3^)–H bonds to prepare thiopyrans **52**.

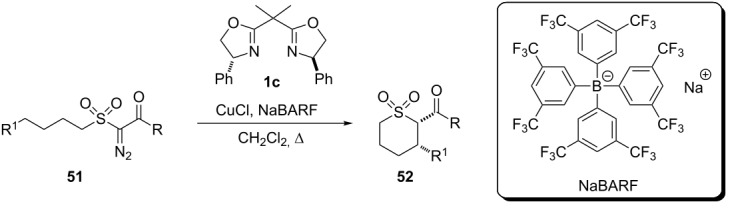					

diazo compound	R	R^1^	time (h)	**52** yield (%)	ee (%)

**51a**	OCH_3_	Ph	5	47	98
**51b**	OCH_3_	4-tolyl	5	64	96
**51c**	OCH_3_	4-anisyl	22	56	91
**51d**	OCH_3_	4-nitrophenyl	2,5	–	–
**51e**	OCH_3_	benzyl	7	42	96
**51f**	OCH_3_	ethyl	16	68	97
**51g**	OBn	octyl	22	66	90
**51h**	CH_3_	Ph	22	30	85
**51i**	Ph	Ph	6	49	97

The authors also performed the copper carbenoid insertion reaction to yield five-membered cyclic sulfones **54**, under similar experimental conditions, in moderate yields and enantiomeric excesses of the *trans* stereoisomer (Table 6).

**Table 6:** Asymmetric insertion of copper carbenoids in C(sp^3^)–H bonds to prepare five-membered cyclic sulfones **54**.

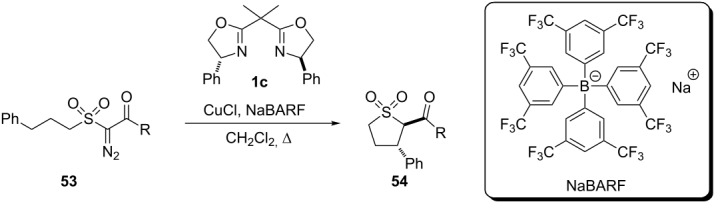				

diazo compound	R	time (h)	**54** yield (%)	ee (%)

**53a**	OCH_3_	5	57	60
**53b**	CH_3_	3	40	40

Independent to the size of the product, the authors emphasize the low dependence of the enantioselectivity with respect to the structural nature of the substrates where the lowest results are observed for the substrate **51h** (R = CH_3_). In contrast, the reaction times showed to be more dependent on the chemical structure of the substrates.

This research group has focused a lot of efforts to better comprehend the scope of this catalytic system, especially on features concerning the BARF salt effect [43–44] and electronic effects on the aromatic rings of the chiral ligands [45].

**Scheme 11:**
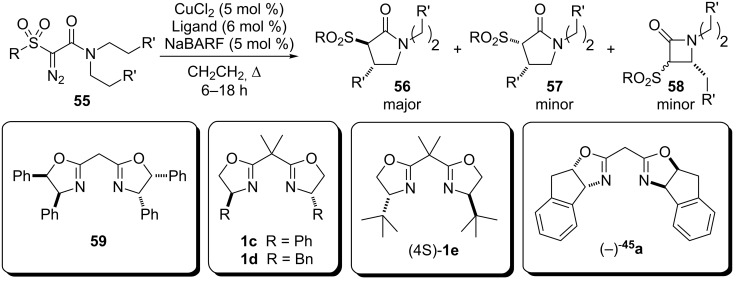
Asymmetric insertion of copper carbenoids in C(sp^3^)–H bonds to prepare *trans*-γ-lactam.

In 2014, Maguire at al reported the syntheses of *N*-heterocycles by the enantioselective insertion of copper carbenoids to α-nitrogen C(sp^3^)–H bonds of amides (Scheme 11) [46]. A wide range of bis(oxazolines) were evaluated as chiral ligands in dry dichloromethane with NaBARF as additive. Three catalytic systems, based on ligands (−)-**45a**, (+)-**45a** and (4*S*)-**1e**, showed a better performance (>82% ee). The transformation was regio and stereoselective where the main product was *trans*-γ-lactam (**56**). The chiral rhodium complexes (*S*)-**17**, (*S*)-**18** and (*S*)-**23** were also evaluated and yielded similar regio- and diastereoselectivity, however, with lower enantioselectivity when compared to the bis(oxazoline)/CuCl_2_/NaBARF catalytic system.

Attempts to heterogeneous catalysis using chiral copper complexes were also done. Fraile et al reported, in 2011, the copper catalyst **60** for enantioselective insertion of carbenoid into *O*-heterocycles C(sp^3^)–H bonds (Table 7) [47–48]. The reaction was performed under homogeneous and heterogeneous conditions, with laponite as support for the catalyst. The reaction afforded moderate yields, diastereomeric ratio and enantioselectivity under both conditions. The supported catalytic system was reused over three cycles with no performance decrease. The same heterogeneous catalyst was also used to perform an enantioselective insertion of the carbenoid into benzylic C(sp^3^)–H bonds and similar results were observed [49].

**Table 7:** Asymmetric insertion of copper carbenoids into tetrahydrofuran C(sp^3^)–H bonds under heterogeneous reaction condition.

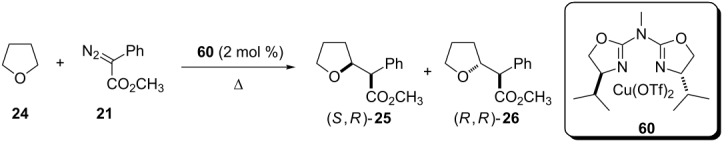			

conditions	% yield	**25**/**26**	**25** ee (%)

homogeneous	74	56:44	64
heterogeneous	50	59:41	62

In 2011, the same research group developed a new heterogeneous copper catalyst for carbenoid insertion into C(sp^3^)–H bonds [50]. The solid support was based on SiO_2_/Al_2_O_3_ and, after addition of ligand **1c** to the reaction media, the reaction afforded moderate yields, diastereomeric ratio and enantioselectivity. These catalysts were reused over three cycles with progressive yield and enantioselectivity decrease.

#### Iridium-based chiral catalysts

**Figure 5:**
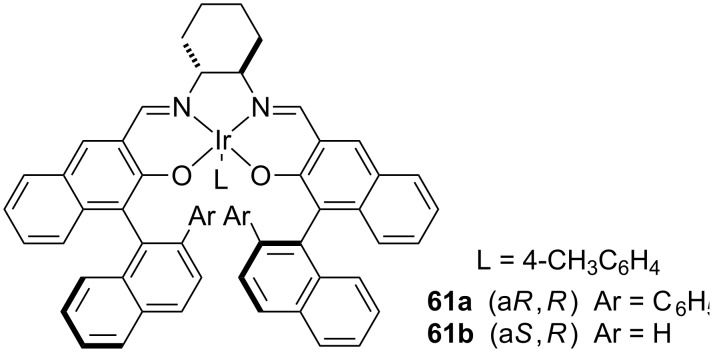
Iridium catalysts used by Suematsu and Katsuki for carbenoid insertion into C(sp^3^)–H bonds.

Most recently, chiral iridium complexes have been used as catalyst for insertion reactions in C(sp^3^)–H bonds. In 2009, Suematsu and Katsuki published the first study addressed to the use of iridium-based chiral complexes as catalyst for the formation of carbenoid intermediates (Figure 5) [51]. The authors conducted insertion reactions in C(sp^3^)–H bonds in a diastereo- and enantioselective manner. For enantioselective insertion reactions the authors tested two specific iridium complexes, **61a** and **61b**.

The authors used a wide range of α-substituted α-diazoacetates for performing insertion into substrates such as tetrahydrofuran (**24**) and 1,4-cyclohexadiene (**62**). Using these two compounds, the authors evaluated the best conditions for carrying out the reaction. When the iridium-catalyzed insertion reaction of carbenoids into tetrahydrofurans C(sp^3^)–H bond was performed at room temperature, the authors reported the formation of dimers of α-substituted α-diazoacetates as the main products of this reaction. This issue was circumvented when low temperatures, −50 °C, were used and the insertion reaction occurred with considerable yields and good enantiomeric excess (Table 8). According to the authors, the low temperature could reduce some type of steric strain on the transition state of the insertion reaction and avoid the dimer formation.

**Table 8:** Intermolecular insertion of chiral iridium carbenoid into THF C(sp^3^)–H bond.

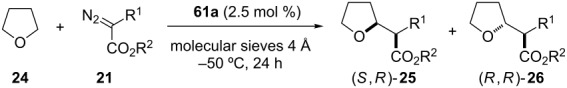					

diazo compound	R^1^	R^2^	**25**:**26**	**25** yield (%)	**25** ee (%)

**21a**	C_6_H_5_	Me	13:1	75	95
**21b**	*p*-MeOC_6_H_4_	Me	>20:1	64	97
**21c**	*p*-ClC_6_H_4_	Me	19:1	82	94
**21d**	*p*-MeC_6_H_4_	Me	19:1	71	97
**21e**	*p*-BrC_6_H_4_	Me	>20:1	76	93
**21f**	*m*-MeOC_6_H_4_	Me	9:1	75	97
**21g**	*m*-ClC_6_H_4_	Me	>20:1	82	95
**21h**	2-naphthyl	Me	>20:1	80	98
**21i**	*o*-MeOC_6_H_4_	Me	>20:1	9	95
**21j**	Me	t-Bu	13:1	70	90

For the insertion reaction of iridium carbenoid into the 1,4-cyclohexadiene (**62**) bis-allylic C(sp^3^)–H bond the authors observed the formation of two products, one resulting from iridium carbenoid insertion into the C(sp^3^)–H bond (**63**) and the other as a result of the cyclopropanation reaction (**64**, Table 9).

**Table 9:** Intermolecular insertion of chiral iridium carbenoids into the 1,4-cyclohexadiene (**62**) bis-allylic C(sp^3^)–H bond.

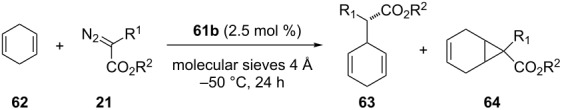					

diazo compound	R^1^	R^2^	**63**:**64**	**63** yield (%)	ee (%) o

**21a**	C_6_H_5_	Me	>20:1	91	94
**21b**	*p*-MeOC_6_H_4_	Me	>20:1	39	90
**21c**	*p*-ClC_6_H_4_	Me	>20:1	79	95
**21f**	*m*-MeOC_6_H_4_	Me	>20:1	95	96
**21g**	*m*-ClC_6_H_4_	Me	>20:1	80	99
**21i**	*o*-MeOC_6_H_4_	Me	>20:1	54	97
**21k**	o-ClC_6_H_4_	Me	>20:1	53	99
**21l**	3,4-Cl_2_C_6_H_3_	Me	>20:1	95	99
**21m**	3-Thienyl	C_2_H_4_Cl	>20:1	67	97
**21n**	Me	Et	>20:1	68	83
**21o**	Me	t-Bu	>20:1	84	>99

Both examples reported by Suematsu and Katsuki showed very good yields and excellent enantiomeric excesses of the products. This work is noteworthy because it is the first report in the literature of an enantioselective insertion of an iridium carbenoid into C(sp^3^)–H bonds.

**Scheme 12:**
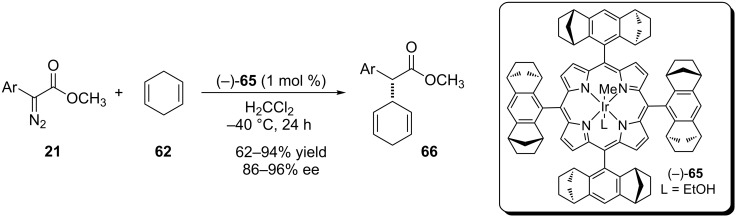
Chiral porphyrin iridium complex catalyzes the carbenoid insertion into bis-allylic C(sp^3^)–H bonds.

Che and coworkers introduced the first porphyrin-based chiral iridium catalyst (−)-**65** to insertion of carbenoids into C(sp^3^)–H bonds [52]. The reaction with 1,4-cyclohexadiene was promoted by 1 mol % of the catalyst at low temperatures to affords the product in high yields and enantioselectivity (Scheme 12).

**Scheme 13:**
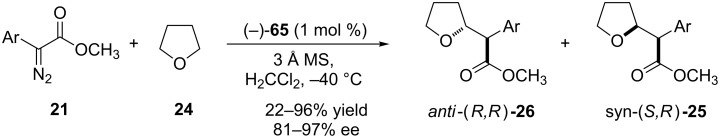
Chiral porphyrin iridium complex catalyzes the carbenoid insertion into tetrahydrofuran C(sp^3^)–H bonds.

The same catalytic system was applied to carbenoid insertions into tetrahydrofuran C(sp^3^)–H bonds (Scheme 13). The reaction afforded the desired product in a regioselective way and high diastereoselectivity, ranging from 2.5:1 to >20:1, favoring the *anti*-product, in a complementary sense when compared to the results reported by Suematsu and Katsuki for iridium catalyst **61a** (Table 8). Poor to excellent yields and high enantioselectivity were reported for the main product.

**Scheme 14:**
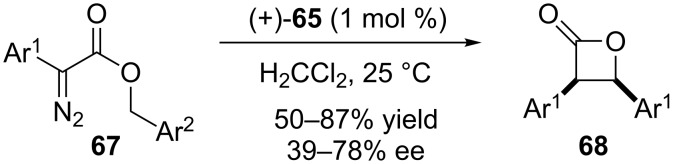
Chiral porphyrin–iridium complex catalyzes the intramolecular carbenoid insertion into C(sp^3^)–H bonds to afford the synthesis of *cis*-β-lactones.

The chiral porphyrin-based iridium complex (−)-**65** was also used by the same research group to catalyze the intramolecular carbenoid insertion into C(sp^3^)–H bonds and affords the synthesis of *cis*-β-lactones in a wide range of yields and enantioselectivities (Scheme 14) [53]. The reaction time was dependent on the chemical structure of the group Ar^1^ (*p*-CH_3_Ph = 24 h; *p*-FPh, *m*-ClPh, *m*-BrPh = 10 min) and the enantioselectivity drops from 70–80% ee to less than 50% ee when Ar^1^ is *m*-ClPh or *m*-BrPh.

**Scheme 15:**
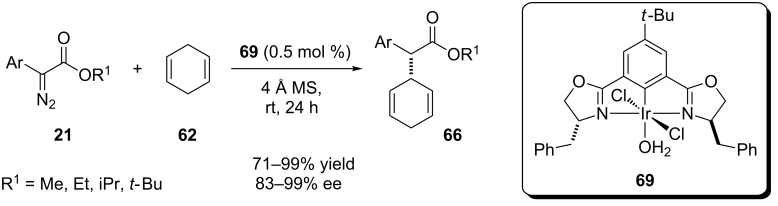
Chiral bis(oxazoline)–iridium complex catalyzes the carbenoid insertion into bis-allylic C(sp^3^)–H bonds.

In 2013, Davies, Blakey and coworkers reported a new iridium catalyst to perform a carbenoid insertion into the C(sp^3^)–H bond (Scheme 15) [54]. The reactions were performed at room temperature and low catalyst loading (0.5 mol %) to afford the desired product in high yield and enantioselectivity. To reduce the amount of cyclohexadiene, the reaction was also performed in trifluorotoluene, resulting in a yield decrease (93% when 1,4-cyclohexadiene was solvent and reagent; 60% when 2,5 equivalents of cyclohexadiene and PhCF_3_ as solvent where used) but with almost the same enantioselectivity.

#### Rhodium-based chiral catalysts

Since the pioneering reports by Ikegami [39], Doyle [40] and Davies [41] introducing their dirhodium chiral catalysts, these complexes have been the most frequently used and studied catalysts for enantioselective insertion of carbenoids into C(sp^3^)–H [55]. It is noteworthy the intensive contribution of the Davies research group which reported important works related to regioselectivity and stereoselectivity [56], and chemoselectivity [57] of this transformation.

**Scheme 16:**
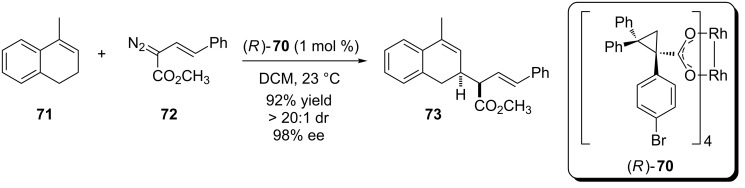
New cyclopropylcarboxylate-based chiral catalyst to enantioselective carbenoid insertion into the endocyclic allylic C(sp^3^)–H bond.

In 2011, Davies et al reported a chiral rhodium complex based on a new cyclopropylcarboxylate ligand (Scheme 16) [58]. Among the various transformations promoted by this new catalyst we can find enantioselective carbenoid insertion into the endocyclic allylic C(sp^3^)–H bond of **71** followed by the Cope rearrangement and retro-Cope strategy previously described by the same research group [59]. The product was obtained in excellent yield, diastereo- and enantioselectivity.

**Scheme 17:**
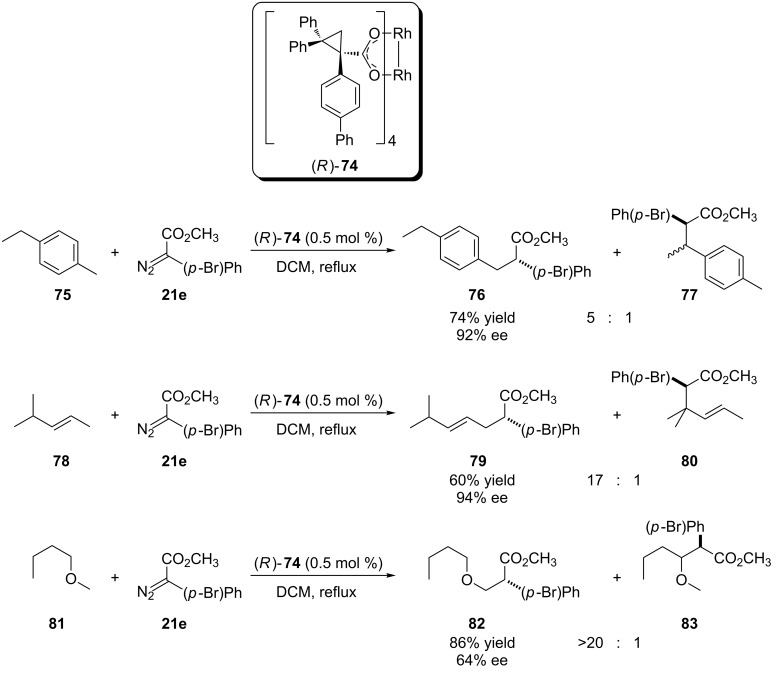
Regio- and enantioselective carbenoid insertion into the C(sp^3^)–H bond catalyzed by a new bulky cyclopropylcarboxylate-based chiral dirhodium complex (*R*)-**74**.

Later, the same authors showed a new chiral rhodium complex (*R*)-**74** based on an analogue cyclopropylcarboxylate ligand (Scheme 17) [60]. This new ligand favors the regiochemistry of rhodium carbenoid insertion into primary C(sp^3^)–H activated bonds even in the presence of activated secondary C(sp^3^)–H bonds. This preference stems from the greater volume of the ligand and the consequent greater steric strain in the transition state that leads to the minor insertion product at activated secondary C(sp^3^)–H bonds. Substrates with benzylic bond, allylic and α-oxygen C(sp^3^)–H were submitted to the new catalyst, under dichloromethane reflux, and led to the preferential formation of the insertion products into primary carbon, (from 5:1 to >20:1), high yields and enantioselectivity, higher than 90% ee, 88% ee and 64% ee, respectively.

**Scheme 18:**
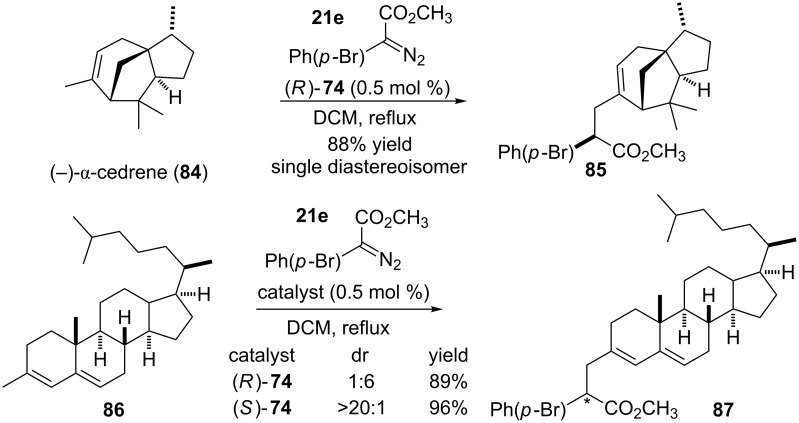
Regio and diastereoselective carbenoid insertion into the C(sp^3^)–H bond catalyzed by a new bulky cyclopropylcarboxylate-based chiral dirhodium complex.

The authors also employed this catalyst in the functionalization of the (−)-α-cedrene and a steroidal nucleus, both substrates containing primary, secondary and tertiary allylic C(sp^3^)–H bonds (Scheme 18). In both cases, was only observed the formation of the regioisomer derived from carbenoid insertion into the primary allylic position with excellent yield and high diastereoselectivity. For the steroidal substrate, the catalyst (*R*)-**74** favored the formation of a new center with *R* configuration in a 6:1 diastereoisomeric ratio. The use of the enantiomeric catalyst, (*S*)-**74**, yielded the product with *S* configuration at the new stereogenic center with a higher diastereoisomeric ratio (>20:1).

**Scheme 19:**
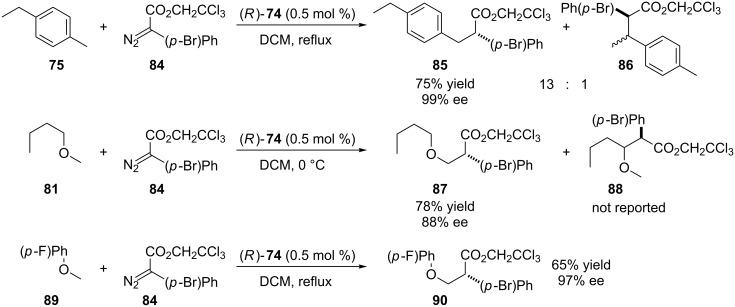
2,2,2-Trichloroethyl (TCE) aryldiazoacetates to improve the scope, regio- and enantioselective of the carbenoid insertion into primary C(sp^3^)–Hs bond by (*R*)-**74**.

In 2014, Davies and coworkers expand the scope of catalyst (*R*)-**74** by combining it with 2,2,2-trichloroethyl (TCE) aryldiazoacetates (Scheme 19) [61]. When compared with the use of traditional methylaryldiazoacetates (Scheme 17), an improved enantioselectivity of the insertion product **85** was observed combined with superior regiochemistry, favoring the rhodium carbenoid insertion into primary C(sp^3^)–H activated bonds even in the presence of activated secondary C(sp^3^)–H bonds.

Another important contribution addressed by this work was the C(sp^3^)–H bond functionalization of methyl ethers even in the presence of other activated C(sp^3^)–H bonds. A wide range of methyl ethers were regioselectively functionalized, also with improved enantioselectivity, by the use of TCE aryldiazoacetates in combination with (*R*)-**47**, here exemplified by the reaction between methyl ether **81** and the TCE aryldiazoacetate **84**. The comparison of this result with that presented at Scheme 17 shows a significant increase of the enantioselectivity.

TCE heteroaryldiazoacetates were also successfully employed for the formation of the rhodium carbenoid insertion products in superior yields when compared to the reaction with methyl aryldiazoacetates. TCE aryldiazoacetates reduced significantly the carbene dimerization allowing the reduction of the TCE diazoacetate addition time from 1.5 hours to 5 seconds.

The deactivated aryl methyl ether **84** was also functionalized by the use of TCE aryldiazoacetates in combination with (*R*)-**47** in good yield and excellent enantioselectivity. The reaction between **84** and the methyl aryldiazoacetate **21e** afforded the insertion product in only 15% yield.

**Scheme 20:**
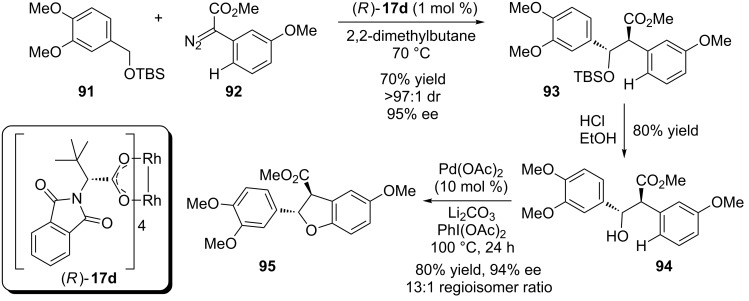
Sequential C–H functionalization approach to 2,3-dihydrobenzofurans.

In 2013, Davies, Yo et al reported a new strategy to construct 2,3-dihydrobenzofurans based on a sequential enantioselective rhodium catalyzed carbenoid insertion into a C(sp^3^)–H bond followed by a palladium C(sp^2^)–H bond activation to build a new C–O bond (Scheme 20) [62]. A wide range of benzyl silyl ethers and diazo compounds were tested providing the desired 2,3-dihydrobenzofuran in good yields and excellent diastereo- and enantioselectivity. Later, this strategy was further used by Davies, Zakarian and coworkers to access the total synthesis of (−)-maoecrystal V [63].

**Scheme 21:**
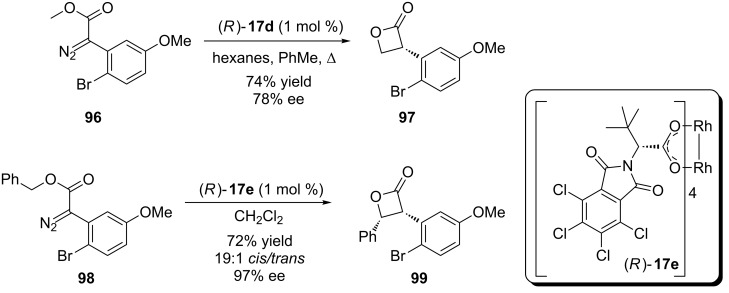
Enantioselective intramolecular rhodium carbenoid insertion into C(sp^3^)–H bonds to afford *cis*-disubstituted β-lactones.

During this study, the authors observed an unexpected result when *ortho*–halosubstituted diazo compounds were used. Here the formation of a β-lactone by the carbenoid insertion into the C(sp^3^)–H bond of the alkyl substituent of the alkoxy moiety of the ester (Scheme 21). The authors decided to investigate this observation and reported a more detailed study concerning the synthesis of *cis*-disubstituted β-lactones in high yield, diastereo- and enantioselectivity [64].

Total syntheses of 2,3-dihydrobenzofurans containing natural products have also been recently reported independently by Hashimoto [65–66] and Kan [67] based on an enantioselective intramolecular rhodium carbenoid insertion into C(sp^3^)–H bonds.

In 2012, Pavlyuk and coworkers performed the synthesis of azacycloalkenes by rhodium carbenoid insertion into C(sp^3^)–H bonds, and subsequent ring closing olefin metathesis (RCM) [68]. The insertion of the rhodium carbenoids derived from vinyl diazoacetate into the C(sp^3^)–H bonds of the alkenylcarbamates **97a–d** yields two reaction products (Table 10). The major one (**99a–d**) was the result of the cyclopropanation reaction of the double bond present in **97a–d**. The minor product (**100a–d**) was the desired one, resulting from the insertion reaction on the C(sp^3^)–H bond α to the nitrogen atom.

**Table 10:** Cyclopropanation/Insertion rhodium carbenoid reactions into C(sp^3^)–H reported by Pavlyuk and coworkers.

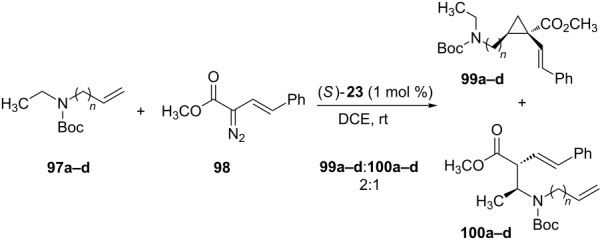							

alkenylcarbamate	*n*	**99** yield (%)	de (%)	ee (%)	**100** yield (%)	de (%)	ee (%)

**97a**	1	64	95	96	32	90	92
**97b**	2	61	98	92	30	94	90
**97c**	3	59	98	95	28	98	85
**97d**	4	55	98	92	27	98	83

The carbenoid insertion reaction into C(sp^3^)–H bonds was regioselective for substrates **97a–d**, even when there was an allylic and α-nitrogen C(sp^3^)–H bond in substrate **97a**. The authors also point out that the **66**:**67** ratio was 2:1 regardless of the rhodium source (Rh_2_(OAc)_4_, Rh_2_(pfb)_4_, Rh_2_(TFA)_4_, Rh_2_(TPA)_4_) or solvents (hexane, benzene) used in this reaction.

The dienes **100a–d** were submitted to 2^nd^-generation Grubbs–Hoveyda catalyst (**101**), under dichloroethane reflux, to afford the desired azacycloalkenes **102a–c** in 95–98% yield and 92–95 % ee (Table 11). Only the diene **100d** did not cyclize and did not afford the nine-membered heterocycle by this methodology.

**Table 11:** Syntheses of *N*-heterocycles by RCM reported by Pavlyuk and coworkers.

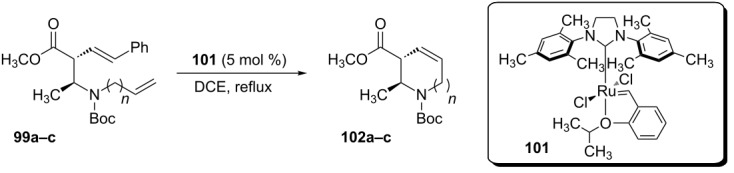				

diene	*n*	**102** yield (%)	de (%)	ee (%)

**99a**	1	98	>98	93
**99b**	2	96	>98	95
**99c**	3	95	>98	92

**Scheme 22:**
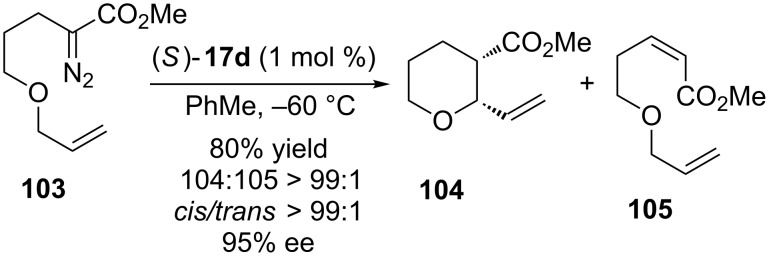
Enantioselective intramolecular rhodium carbenoid insertion into C(sp^3^)–H bonds to afford *cis*-2-vinyltetrahydropyran-3-carboxylates.

In 2015, Hashimoto et al reported the synthesis of methyl 2-vinyltetrahydropyran-3-carboxylates (**104**) by an enantioselective rhodium carbenoid insertion into C(sp^3^)–H bond strategy [69]. The desired product was obtained in very good yield and excellent diastereo- and enantioselectivity favoring the *cis* isomer (Scheme 22).

**Scheme 23:**
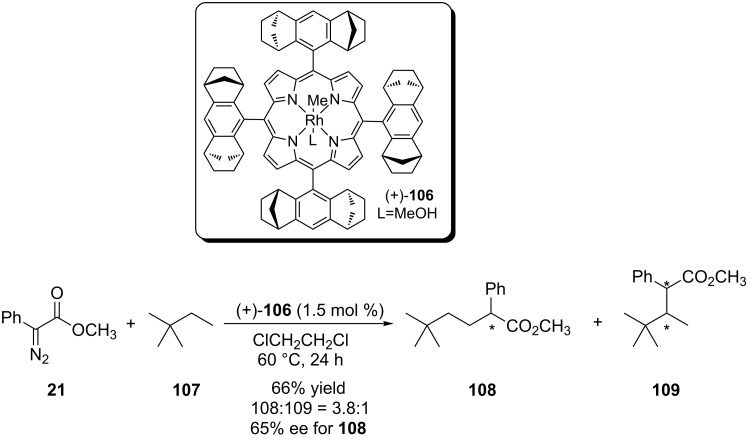
First rhodium porphyrin-based catalyst for enantioselective carbenoid insertion into C(sp^3^)–H bond.

An interesting work was reported by Che and coworkers concerning the first rhodium porphyrin-based catalyst for enantioselective carbenoid insertion into C(sp^3^)–H bonds [70]. The reaction with acyclic alkanes showed regioselectivity in favor of the formation of the insertion product into primary carbons in modest stereoselectivity (Scheme 23). The preference for the reaction in less hindered carbon was attributed to high steric demand required by the chiral ligand in the transition state of the carbenoid insertion step in the C(sp^3^)–H bond.

**Scheme 24:**
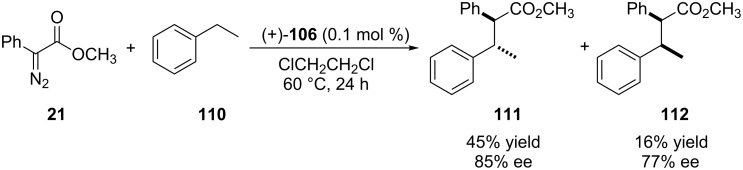
Rhodium porphyrin-based catalyst for enantioselective carbenoid insertion into benzylic C(sp^3^)–H bond.

Cyclic alkanes were also tested with yields ranging from 64–80% and enantioselectivities between 88 and 92% ee. The reaction with cyclohexane was conducted on a gram scale and, after 10 hours of reaction, 2.88 g (73% yield) were obtained of the carbenoid insertion product in 91% ee. Cyclohexene showed high regioselectivity for the carbenoid insertion of the allylic C(sp^3^)–H bond, 43% yield, 71% ee and a 60:40 diastereoisomeric ratio. The formation of the cyclopropanation product was also observed in 14% yield. Ethylbenzene (**110**) was used and also showed high regioselectivity favoring the carbenoid insertion into benzylic C(sp^3^)–H bonds (Scheme 24). The diastereoisomers **111** and **112** were obtained in 45% yield and 16%, respectively, and moderate stereoselectivity was observed in both products.

Few examples of attempts to develop heterogeneous catalytic systems based on chiral rhodium complexes were also done. In 2010, Hashimoto and coworkers reported the synthesis of a highly robust polymer-supported chiral dirhodium(II) complex [71]. The chiral part of the catalyst was based on the *N*-phthaloyl-(S)-amino rhodium carboxylate (*S*)-**17d**. Two substrates were submitted to the enantioselective carbenoid insertion into the C(sp^3^)–H bond in toluene as solvent and at temperatures ranging from −78 °C to −60 °C. The desired products were obtained in yields up to 80% and enantioselectivity up to 90%, even after 15 recycles of the catalyst.

Jones, Davies and coworkers also recently published a new heterogeneous catalytic cycle base on homogeneous catalyst (*S*)-**23** [72]. The chiral scaffold was covalently supported on silica surface through an organic linker and was tested in a tandem enantioselective carbenoid insertion into C(sp^3^)–H bond/Cope rearrangement. The reactions afforded good yields and excellent enantioselectivity. The recycling of the catalyst was evaluated in a cyclopropanation reaction and no significant decrease on its performance could be observed after five runs.

## Conclusion

The efforts focused on the development of the enantioselective insertion of carbenoids into C(sp^3^)–H bonds have provided a wide range of catalytic systems to the chemical community able to perform this transformation and to introduce new C–C bonds in a enantiocontrolled way. The chiral rhodium catalysts are the state of art of this synthetic tool. However, rhodium is an expensive metal and increases the cost of the chemical process despite the low catalyst loads found in literature.

Despite the good results presented until today, the use of the reported chiral iridium catalysts is even more expensive than the use of other metals such as rhodium, copper and ruthenium, for example. Efforts should be directed toward the development of simpler ligands specially those based on inexpensive chiral building blocks like amino acids and sugars.

The examples of works focused on copper-based catalysts are growing in number and quality. Among the well-known metals able to react with diazo compounds to afford carbenoid intermediates, copper is inexpensive and has a wide range of well-established chiral ligands able to be tested and to inspire the rational design of new ligands.

For all metals commonly used in this transformation, more efforts should be focused towards the development of new and robust heterogeneous catalytic systems. This strategy can reduce the costs related to metals like rhodium or iridium and can also contribute to reduce the disposal of these metals in the environment.

## References

[1] Doyle M P, Duffy R, Ratnikov M, Zhou L (2010). Chem Rev.

[2] Davies H M L, Beckwith R E J (2003). Chem Rev.

[3] Yates P (1952). J Am Chem Soc.

[4] Taber D F, Petty E H (1982). J Org Chem.

[5] Mbuvi H M, Woo L K (2008). Organometallics.

[6] Maas G, Werle T, Alt M, Mayer D (1993). Tetrahedron.

[7] Kubo T, Sakaguchi S, Ishii Y (2000). Chem Commun.

[8] Woo L K, Smith D A (1992). Organometallics.

[9] Müller P, Boléa C (2002). Helv Chim Acta.

[10] Flynn C J, Elcoate C J, Lawrence S E, Maguire A R (2010). J Am Chem Soc.

[11] Slattery C N, Maguire A R (2013). Tetrahedron Lett.

[12] Nakamura E, Yoshikai N, Yamanaka M (2002). J Am Chem Soc.

[13] Ye T, García F C, McKervey M A (1995). J Chem Soc, Perkin Trans 1.

[14] Ye T, McKervey M A, Brandes B D, Doyle M P (1994). Tetrahedron Lett.

[15] Kennedy M, McKervey M A, Maguire A R, Roos G H P (1990). J Chem Soc, Chem Commun.

[16] McKervey M A, Ye T (1992). J Chem Soc, Chem Commun.

[17] Roos G H P, McKervey M A (1992). Synth Commun.

[18] Doyle M P (1986). Chem Rev.

[19] Kitagaki S, Anada M, Kataoka O, Matsuno K, Umeda C, Watanabe N, Hashimoto S-i (1999). J Am Chem Soc.

[20] Doyle M P, Winchester W R, Hoorn J A A, Lynch V, Simonsen S H, Ghosh R (1993). J Am Chem Soc.

[21] Timmons D J, Doyle M P (2001). J Organomet Chem.

[22] Doyle M P, Ren T, Karlin K D (2001). The Influence of Ligands on Dirhodium(II) on Reactivity and Selectivity in Metal Carbene Reactions. Progress in Inorganic Chemistry.

[23] Doyle M P (1999). Russ Chem Bull.

[24] McCarthy N, McKervey M A, Ye T, McCann M, Murphy E, Doyle M P (1992). Tetrahedron Lett.

[25] Pirrung M C, Zhang J (1992). Tetrahedron Lett.

[26] Estevan F, Herbst K, Lahuerta P, Barberis M, Pérez-Prieto J (2001). Organometallics.

[27] Estevan F, Lahuerta P, Pérez-Prieto J, Sanaú M, Stiriba S-E, Ubeda M A (1997). Organometallics.

[28] Taber D F, Malcolm S C, Bieger K, Lahuerta P, Sanaú M, Stiriba S-E, Pérez-Prieto J, Monge M A (1999). J Am Chem Soc.

[29] Estevan F, Lahuerta P, Pérez-Prieto J, Pereira I, Stiriba S-E (1998). Organometallics.

[30] Lahuerta P, Pereira I, Pérez-Prieto J, Sanaú M, Stiriba S-E, Taber D F (2000). J Organomet Chem.

[31] Davies H M L, Denton J R (2009). Chem Soc Rev.

[32] Giri R, Shi B-F, Engle K M, Maugel N, Yu J-Q (2009). Chem Soc Rev.

[33] Ledon H, Linstrumelle G, Julia S (1973). Tetrahedron Lett.

[34] Taber D F, Petty E H, Raman K (1985). J Am Chem Soc.

[35] Taber D F, Ruckle R E (1986). J Am Chem Soc.

[36] Sonawane H R, Bellur N S, Ahuja J R, Kulkarni D G (1991). J Org Chem.

[37] Doyle M P, Westrum L J, Wolthuis W N E, See M M, Boone W P, Bagheri V, Pearson M M (1993). J Am Chem Soc.

[38] Nozaki H, Moriuti S, Takaya H, Noyori R (1966). Tetrahedron Lett.

[39] Hashimoto S-i, Watanabe N, Ikegami S (1990). Tetrahedron Lett.

[40] Doyle M P, Van Oeveren A, Westrum L J, Protopopova M N, Clayton T W (1991). J Am Chem Soc.

[41] Davies H W L, Hansen T (1997). J Am Chem Soc.

[42] Hansen J, Autschbach J, Davies H M L (2009). J Org Chem.

[43] Slattery C N, Clarke L-A, O’Neill S, Ring A, Ford A, Maguire A R (2012). Synlett.

[44] Slattery C N, Clarke L-A, Ford A, Maguire A R (2013). Tetrahedron.

[45] Slattery C N, O’Keeffe S, Maguire A R (2013). Tetrahedron: Asymmetry.

[46] Clarke L A, Ring A, Ford A, Sinha A S, Lawrence S E, Maguire A R (2014). Org Biomol Chem.

[47] Fraile J M, García J I, Mayoral J A, Roldán M (2007). Org Lett.

[48] Fraile J M, López-Ram-de-Viu P, Mayoral J A, Roldán M, Santafé-Valero J (2011). Org Biomol Chem.

[49] Fraile J M, Mayoral J A, Muñoz A M, Santafé-Valero J (2013). Tetrahedron.

[50] Fraile J M, Mayoral J A, Ravasio N, Roldán M, Sordelli L, Zaccheria F (2011). J Catal.

[51] Suematsu H, Katsuki T (2009). J Am Chem Soc.

[52] Wang J-C, Xu Z-J, Guo Z, Deng Q-H, Zhuo C-Y, Wan X-L, Che C-M (2012). Chem Commun.

[53] Wang J-C, Zhang Y, Xu Z-J, Lo V K-Y, Che C-M (2013). ACS Catal.

[54] Owens C P, Varela-Álvarez A, Boyarskikh V, Musaev D G, Davies H M L, Blakey S B (2013). Chem Sci.

[55] Ford A, Miel H, Ring A, Slattery C N, Maguire A R, McKervey M A (2015). Chem Rev.

[56] Davies H M L, Morton D (2011). Chem Soc Rev.

[57] Nadeau E, Ventura D L, Brekan J A, Davies H M L (2010). J Org Chem.

[58] Qin C, Boyarskikh V, Hansen J H, Hardcastle K I, Musaev D G, Davies H M L (2011). J Am Chem Soc.

[59] Davies H M L, Lian Y (2012). Acc Chem Res.

[60] Qin C, Davies H M L (2014). J Am Chem Soc.

[61] Guptill D M, Davies H M L (2014). J Am Chem Soc.

[62] Wang H, Li G, Engle K M, Yu J-Q, Davies H M L (2013). J Am Chem Soc.

[63] Lu P, Mailyan A, Gu Z, Guptill D M, Wang H, Davies H M L, Zakarian A (2014). J Am Chem Soc.

[64] Fu L, Wang H, Davies H M L (2014). Org Lett.

[65] Natori Y, Tsutsui H, Sato N, Nakamura S, Nambu H, Shiro M, Hashimoto S (2009). J Org Chem.

[66] Ito M, Namie R, Krishnamurthi J, Miyamae H, Takeda K, Nambu H, Hashimoto S (2014). Synlett.

[67] Wakimoto T, Miyata K, Ohuchi H, Asakawa T, Nukaya H, Suwa Y, Kan T (2011). Org Lett.

[68] McMills M C, Humes R J, Pavlyuk O M (2012). Tetrahedron Lett.

[69] Ito M, Kondo Y, Nambu H, Anada M, Takeda K, Hashimoto S (2015). Tetrahedron Lett.

[70] Thu H-Y, Tong G S-M, Huang J-S, Chan S L-F, Deng Q-H, Che C-M (2008). Angew Chem, Int Ed.

[71] Takeda K, Oohara T, Anada M, Nambu H, Hashimoto S (2010). Angew Chem, Int Ed.

[72] Chepiga K M, Feng Y, Brunelli N A, Jones C W, Davies H M L (2013). Org Lett.

